# Individual and combined effects of herbicide tribenuron-methyl and fungicide tebuconazole on soil earthworm *Eisenia fetida*

**DOI:** 10.1038/s41598-018-21288-y

**Published:** 2018-02-14

**Authors:** Jiqiang Chen, Muhammad Saleem, Caixia Wang, Wenxing Liang, Qingming Zhang

**Affiliations:** 1 College of Plant Health and Medicine, Qingdao Agricultural University, Qingdao, Shandong 266109 China; 2 Department of Plant and Soil Sciences, University of Kentucky, Lexington, KY 40546-0312 USA

## Abstract

Earthworms are soil engineers that alter the soil bio-physical properties to favor plant growth whereas pesticides represent a significant threat to their abundance and soil health. Thus, we investigated the toxic effects of tribenuron-methyl (TBM) and tebuconazole (TEB) on the soil earthworm, *Eisenia fetida*. The TBM demonstrated low toxicity to *E*. *fetida* in the contact filter paper and artificial soil tests, with median lethal concentrations (LC_50_) of 135.6 μg cm^−2^ at 48 h and 511 mg kg^−1^ on day 14, respectively. Similarly, TEB also showed low toxicity to *E*. *fetida* in the artificial soil test with LC_50_ of 287 mg kg^−1^ on day 14. However, TEB was highly toxic to earthworm in the contact filter paper test with LC_50_ of 5.7 μg cm^−2^ at 48 h. The mixture of two pesticides had an antagonistic effect on the earthworm. Under 0.1 LC_50_ of TBM and TEB, either single or combined application of pesticides induced oxidative stress and inhibited cellulase activity in early days of the earthworm exposure. However, both pesticides did not damage the earthworm DNA. Our results suggest that pesticides can negatively affect soil earthworms and provide valuable information regarding the responses of soil biological engineers to the lethal agrochemicals.

## Introduction

The use of pesticides in agriculture is a common practice to protect crops all over the world. However, despite their impacts on target species, pesticides potentially affect soil macro- and microorganisms^1,2^. This is because only 0.1% of an applied pesticide reaches the target organism whereas rest of it pollutes the surrounding air, water, and soil environments by different means^3–6^. Earthworms are one of the most common soil invertebrate organisms and considered as soil biophysical engineers. They play an important role in improving the soil biophysical conditions such as soil structure, nutrient immobilization, microbial community structure and functioning to support plant growth and development^7,8^. As compared to other soil animals, earthworms are highly susceptible and sensitives to the soil pollutants. Therefore, these are also widely used as model organisms in ecotoxicological studies to predict the impact of chemicals on soil biodiversity^1,5,6^.

Although a number of studies have reported the impacts of various pesticides on earthworms, yet past research has mainly focused on understanding earthworm responses to the single pesticide. However, in fact, pesticides in the agricultural soils rarely occur as single individual compounds, but rather, exist as mixtures with other chemicals including pesticides^9,10^. Particularly, their occurrence with the other pesticides in soil may change and/or amplify their toxicity^4^, therefore, the results from single pesticide exposures studies may not reflect real toxicological responses of earthworms when exposed to multiple pesticides in the field. Moreover, as per the opinion of Stepić *et al*.^5^, toxcity of a single pesticide may also underestimate the ecological risks of chemicals that exist as mixture in the soil environment. Therefore, it is deemed necessary to investigate and evaluate the ecological risk of pesticides, when applied alone or as mixture on the soil organisms including earthworms.

In a wheat field, herbicide and fungicide are frequently used to control the damage of weed and disease for enhancing crop protection and production. The tribenuron methyl (TBM) [methyl 2-[4-methoxy-6-methyl-1,3,5-triazin-2-yl (methyl) carbamoyl sulfamoyl] benzoate] and tebuconazole (TEB) [(RS)-1-p-chlorophenyl-4, 4-dimethyl-3-(1H-1,2,4-triazol-1-ylmethyl) pentan-3-ol] are two frequently used herbicide and fungicide, which play important roles in controlling the weeds and diseases in wheat field^11,12^. For TBM, it has been widely reported that the yield of some sensitive crops was reduced in agricultural rotation systems due to its phytotoxicity^13–15^. Apart from its phytotoxic effects, there has been an increased interest of researchers in the field to investigate the impact of TBM on soil and environmental health^16,17^. But in case of the TEB, previous studies have shown a relative longer half-life (49–610 d) of this pesticide in the different soil environments. Owing to its longer persistence in soil, TEB negatively affects the soil biological properties^18–20^. Despite the individual effects of TBM and TEB on soil health indicators are studied, their combined effects on soil earthworms remain unknown.

Reactive oxygen species (ROSs) determine the normal functioning of physiological processes within cell environment in the living organisms including earthworms. The ROSs mostly interacts with all types of biological molecules, and cells have ability to neutralize them by the induction of antioxidative enzymes such as superoxide dismutase (SOD), catalase (CAT), etc., or some endogenous antioxidant molecules such as glutathione^21,22^. If the balance between generation and neutralization of ROSs is broken, then oxidative stress may occur in the meantime. Soon after, the overproduction of ROSs may cause damage to the macromolecules such as protein carbonylation, lipid peroxidation, and DNA^23–25^. Apart from the oxidative damage, soil contaminants also negatively affect a vital enzyme called cellulase in earthworm, which plays an important role in the processing of organic matter in its gut^26–28^. The aforementioned biomarkers such as enzymes and DNA damage have been studied to evaluate the effects of contaminants on earthworms in some previous studies^27,28^.

In this study, we investigated the acute and chronic toxicity of both TBM and TEB (alone or combined) on enzyme activity, lipid peroxidation, and DNA damage in a soil earthworm (*Eisenia fetida*). We hypothesize that the earthworm will exhibit differential responses to both pesticides when used alone or as mixture, and earthworm physiological responses would predict the toxicological impacts of these pesticides on soil organisms. This work may provide a comprehensive understanding of the individual and combined effects of pesticides on soil organisms in the contexts of ecological risk assessment and future use of pesticides in agriculture.

## Results

### Acute toxicity of individual and combined pesticides on earthworms

In filter paper contact test, the LC_50_ values of TBM on earthworms were 377.9 and 135.6 μg cm^−2^ at 24 and 48 h, respectively (Table 1). The acute toxicity of TEB was much higher than that of TBM, wherein the LC_50_ values were 9.4 and 5.7 μg cm^−2^ at 24 and 48 h, respectively. In artificial soil test, the LC_50_ values of TBM and TEB were 1007.3 and 746.3 mg kg^−1^ on day 7, respectively. The LC_50_ values of TBM and TEB were 511.0 and 287.9 mg kg^−1^ on day 14, respectively. In pesticides mixture exposure experiment, the LC_50_ of TBM and TEB to earthworms were 114.2, and 4.7 μg cm^−2^ at 48 h in filter paper contact test, respectively. The LC_50_ of TBM and TEB were 479.8 and 247.9 mg kg^−1^ in artificial soil test at 48 h, respectively. The AI values in both toxic tests at different exposure time were −0.44, −0.67, −0.56, and −0.80, indicated an antagonistic effect between TBM and TEB.

**Table 1:** Individual and combined acute toxicity assessments with contact filter paper and artificial soil tests.

Toxicity method	Time	Individual toxicity LC_50_(95% confidence interval)		Combined toxicity (1:1 toxicity) LC_50_(95% confidence interval)		Additive index	Combined action
		Tribenuron-methyl	Tebuconazole	Tribenuron-methyl	Tebuconazole		
Contact filter paper test (μg cm^−2^)	24 h	377.9 (195.6–526.3)	9.4 (8.5–10.6)	202.8 (168.7–251.0)	8.5 (7.1–10. 5)	−0.44	antagonism
	48 h	135.6 (103.4–150.6)	5.7 (3.7–7.6)	114.2 (94.8–141.6)	4.7 (3.9–5.9)	−0.67	antagonism
Artificial soil test (mg kg^−1^)	7 d	1007.3 (898.7–1235.6)	746.3 (690.3–806.5)	926.7 (802.8–1091)	478.7 (414.5–563.6)	−0.56	antagonism
	14 d	511.0 (401.5–623.3)	287.9 (119.4–415.1)	479.8 (242.1–560.3)	247.9 (125.1–367.4)	−0.80	antagonism

### Effects of individual and combined pesticides on enzyme activities

**Figure 1:**
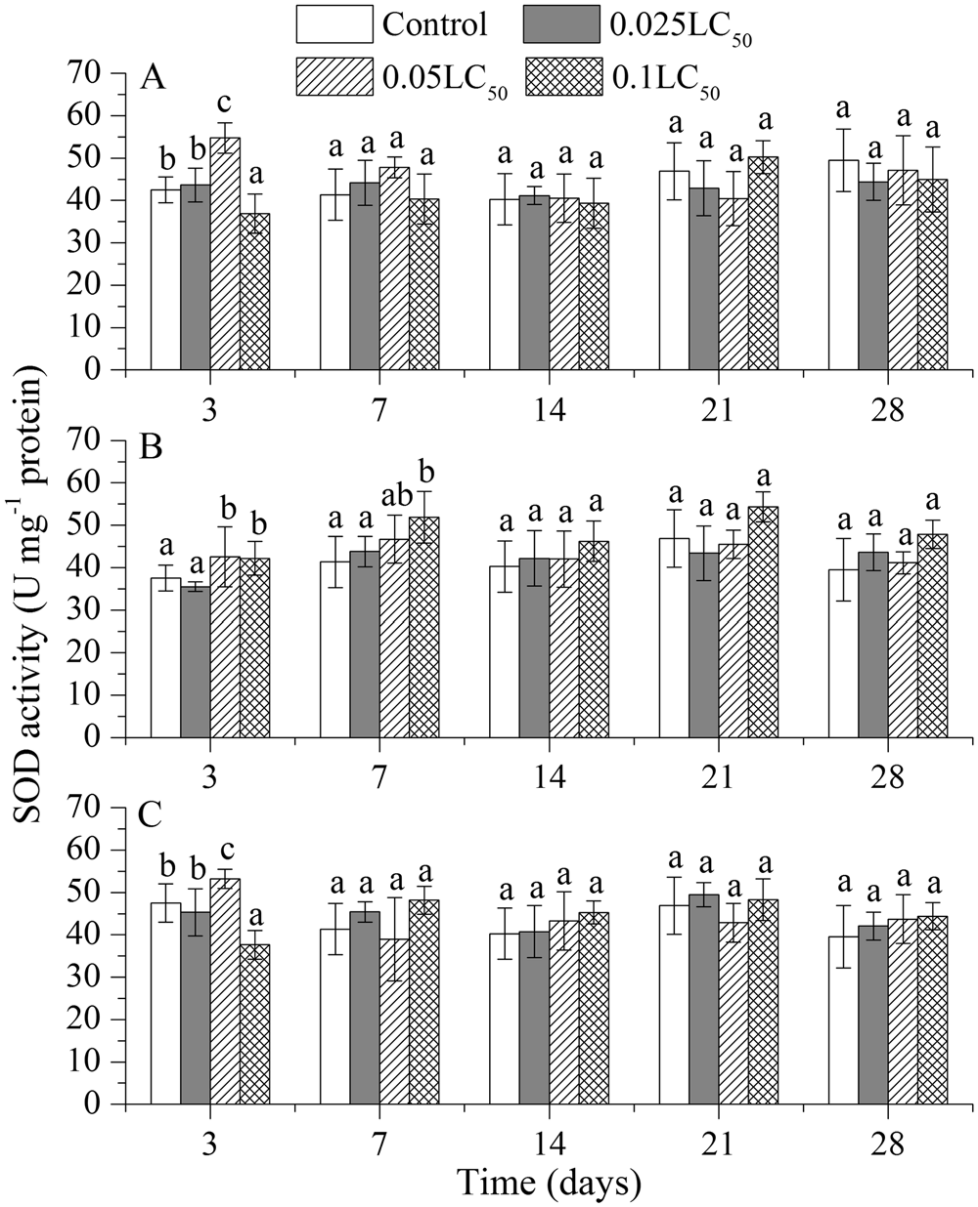
Effects of tribenuron-methyl (**A**), tebuconazole (**B**), and mixture of both pesticides (**C**) on superoxide dismutase (SOD) activity in *E*. *fetida*. Each column represents the mean of three replicates, and the error bars represent the standard deviations (SD). Different letters above bars are significantly different at the confidence level of 0.05 between treatments at the same time.

Only on day 3, the SOD activity was significantly stimulated in earthworms exposed to medium concentration (0.05 LC_50_) of TBM but was significantly inhibited by high concentration of (0.1 LC_50_) TBM as compared to the control treatments (Fig. 1A). After 3 days, there was no obvious difference in the SOD activities of earthworms in controls and pesticides treatments. In case of TEB, under medium and high concentration, the SOD activity was relatively higher than control during the first 7 days (Fig. 1B). Under combined exposure of TBM and TEB, the changes in SOD activity were very likely due the interaction of TBM and TEB in pesticides mixture. The SOD activity was significant effected by medium and high concentrations of both pesticides only on day 3 (Fig. 1C).

**Figure 2:**
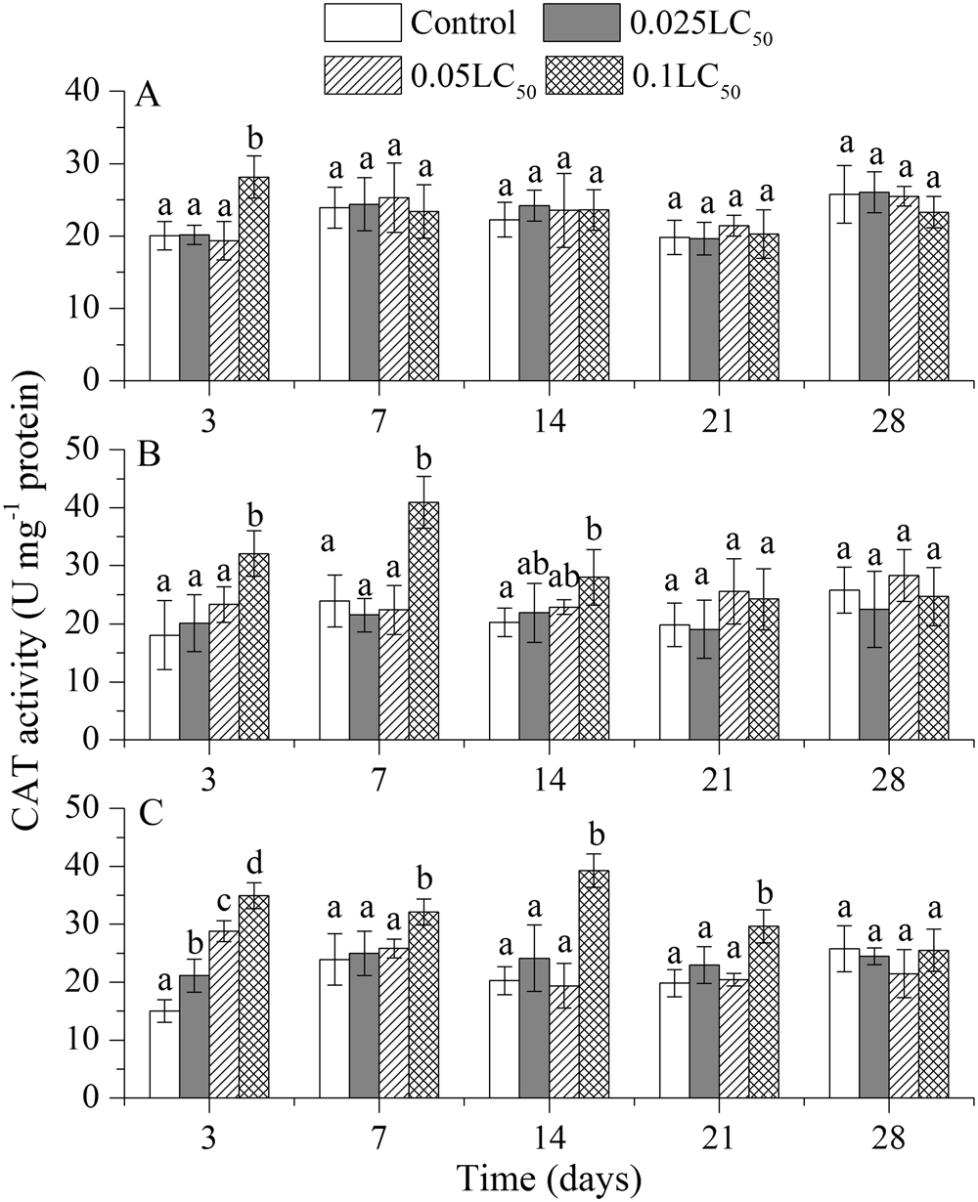
Effects of tribenuron-methyl (**A**), tebuconazole (**B**), and mixture of both pesticides (**C**) on catalase (CAT) activity in *E*. *fetida*. Each column represents the mean of three replicates, and the error bars represent the standard deviations (SD). Different letters above bars are significantly different at the confidence level of 0.05 between treatments at the same time.

The changes in CAT activity were described in Fig. 2. During whole exposure period, the CAT activity increased significantly as compared to control treatment under high concentration of TBM only on day 3 (Fig. 2A). The stimulation effect of a high concentration of TEB on earthworm was longer than that of TBM, which continued until day 14 (Fig. 2B). Under combined use of TBM and TEB, the stimulation of earthworm increased with increasing doses of pesticides mixture, indicating a dose-effect relationship on day 3. Overall on day 3, only high concentrations of combined TBM and TEB stimulated the CAT activity compared to the control treatments (Fig. 2C). On last day of exposure (28 d), all pesticides treatments differed with control treatments in the earthworm stimulation.

**Figure 3:**
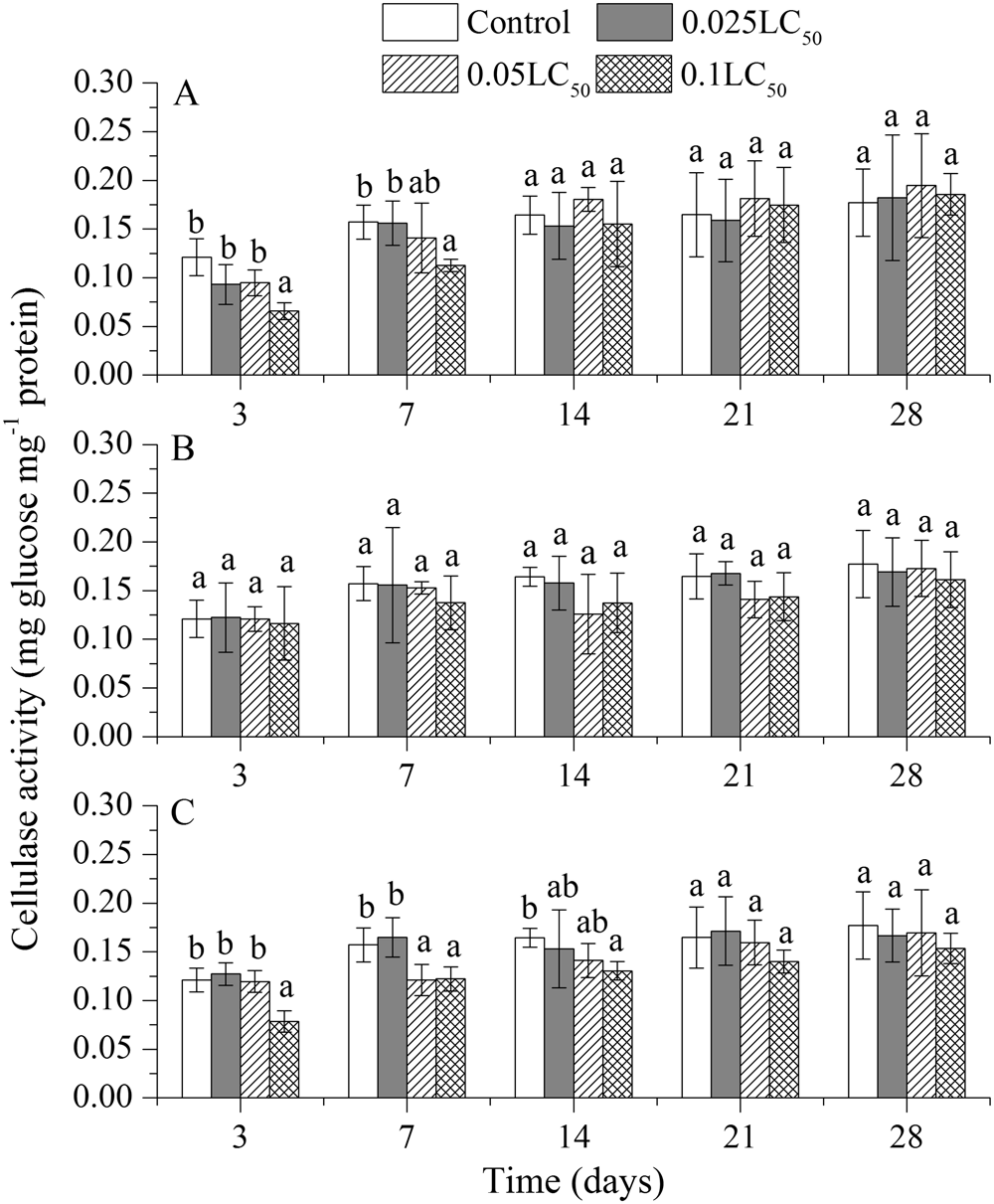
Effects of tribenuron-methyl (**A**), tebuconazole (**B**), and mixture of both pesticides (**C**) on cellulase activity in *E*. *fetida*. Each column represents the mean of three replicates, and the error bars represent the standard deviations (SD). Different letters above bars are significantly different at the confidence level of 0.05 between treatments at the same time.

We also determined differences in cellulase activity in the earthworms exposed to individual and combined applications of TBM and TEB (Fig. 3). The cellulase activity was inhibited at only high concentration of TBM on day 3 and 7 compared to the control treatments (Fig. 3A). The cellulase activity did not differ in control and TEB exposure experiments during the whole period (Fig. 3B). Under high doses of pesticides mixture, the cellulase activity was significantly lower than that of control in the first 14 days, and then it recovered to the control level (Fig. 3C).

### Effects of individual and combined pesticides on lipid peroxidation and DNA damage

**Figure 4:**
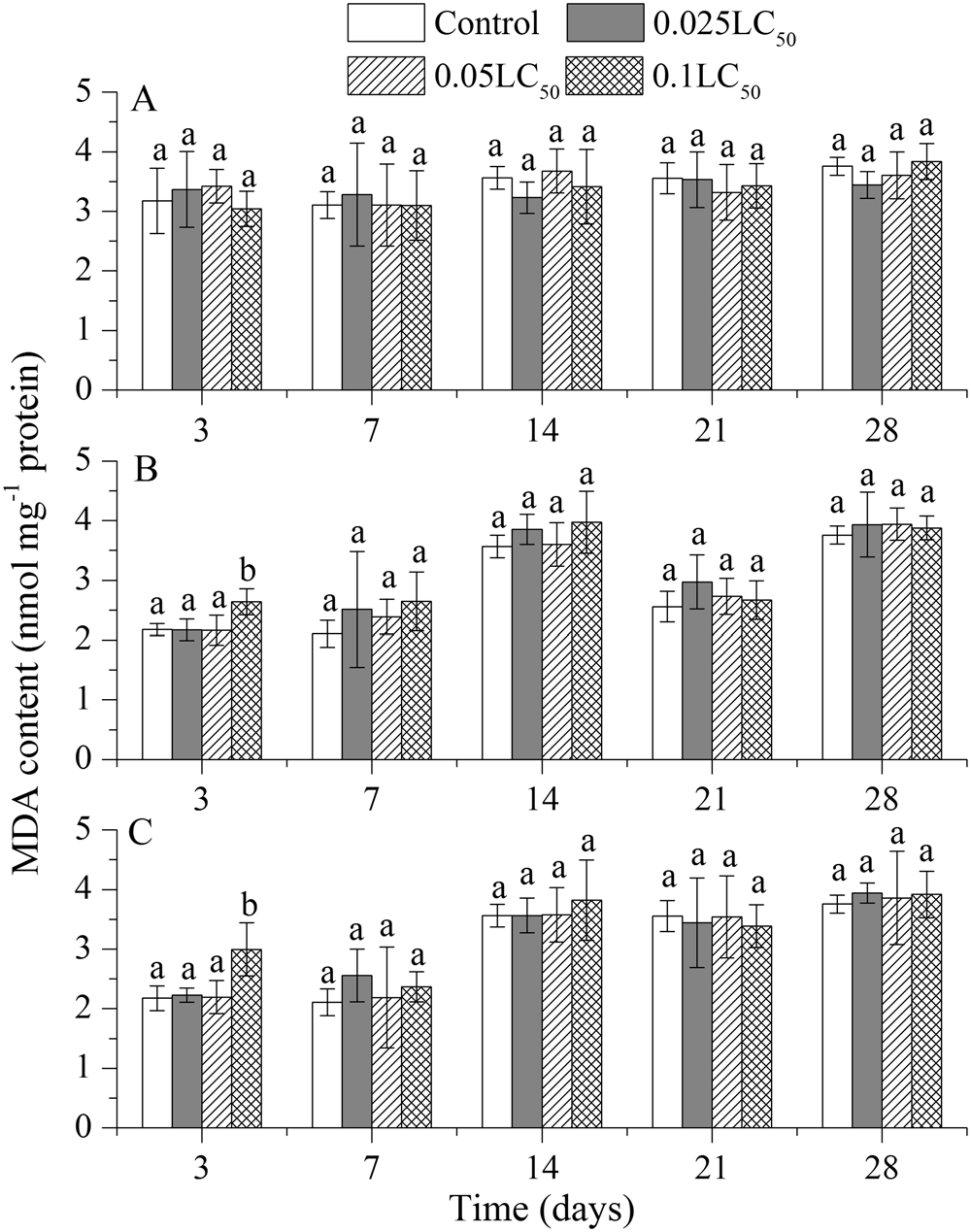
Effects of tribenuron-methyl (**A**), tebuconazole (**B**), and mixture of both pesticides (**C**) on malondialdehyde (MDA) content in *E*. *fetida*. Each column represents the mean of three replicates, and the error bars represent the standard deviations (SD). Different letters above bars are significantly different at the confidence level of 0.05 between treatments at the same time.

As shown in Fig. 4A, only on day 3, the MDA content in earthworms treated with higher concentration of TEB was significant higher than that of the control treatments. After three days, the MDA contents in all treatments recovered to control levels (Fig. 4B). The changes in MDA contents were almost same in the individual TEB and mixture treatments. Only on day 3, the MDA contents were relatively high in pesticides mixture treatments than that of the control (Fig. 4C).

Finally, we investigated the individual and combined effects of pesticides on earthworm DNA damage (Table 2). Interestingly, pesticides used, alone or as mixture, did not cause significant damage to the earthworm DNA than that of control.

**Table 2:** Comet assay results of coelomocytes in *E*. *fetida* exposed to tribenuron-methyl, tebuconazole and the mixture of both pesticides (mean ± SD). The same letters in the columns indicate no significant difference between treatments at the same time (*p* < 0.05).

Pesticide	Dose (mg kg^−1^)	Length of Comet (μm)					Olive Tail Moment (OTM)				
		3	7	14	21	28 d	3	7	14	21	28 d
Tribenuron-methyl	0	3.04 ± 0.73a	3.21 ± 1.02a	2.98 ± 0.85a	3.64 ± 0.92a	3.33 ± 1.14a	0.74 ± 0.20a	0.56 ± 0.14a	0.44 ± 0.20a	0.46 ± 0.17a	0.65 ± 0.23a
	0.025LC_50_	3.32 ± 0.2a	2.54 ± 0.65a	3.3 ± 0.92a	2.76 ± 0.84a	3.5 ± 1.23a	0.65 ± 0.17a	0.54 ± 0.15a	0.39 ± 0.12a	0.72 ± 0.22a	0.55 ± 0.15a
	0.05LC_50_	2.78 ± 0.8a	3.03 ± 0.85a	2.88 ± 0.64a	3.54 ± 1.04a	3.2 ± 0.8a	0.71 ± 0.28a	0.62 ± 0.16a	0.58 ± 0.17a	0.55 ± 0.15a	0.62 ± 0.18a
	0.1LC_50_	3.54 ± 1.13a	3.05 ± 0.76a	3.3 ± 0.95a	3.2 ± 0.8a	3.23 ± 0.68a	0.59 ± 0.14a	0.75 ± 0.20a	0.53 ± 0.15a	0.62 ± 0.18a	0.53 ± 0.12a
Tebuconazole	0	2.54 ± 0.85a	2.65 ± 0.85a	3.05 ± 0.54a	2.85 ± 0.81a	3.1 ± 0.88a	0.72 ± 0.15a	0.67 ± 0.20a	0.58 ± 0.23a	0.64 ± 0.22a	0.56 ± 0.16a
	0.025LC_50_	2.88 ± 0.6a	3.04 ± 0.80a	2.89 ± 1.13a	3.24 ± 0.68a	2.86 ± 0.64a	0.68 ± 0.16a	0.53 ± 0.12a	0.70 ± 0.15a	0.58 ± 0.12a	0.63 ± 0.14a
	0.05LC_50_	2.83 ± 0.76a	2.98 ± 0.56a	3.32 ± 0.78a	3.07 ± 0.78a	3.22 ± 0.59a	0.53 ± 0.17a	0.61 ± 0.13a	0.58 ± 0.14a	0.63 ± 0.10a	0.65 ± 0.10a
	0.1LC_50_	3.18 ± 0.75a	3.34 ± 0.95a	3.24 ± 0.74a	3.35 ± 0.90a	3.05 ± 0.0.57a	0.78 ± 0.22a	0.70 ± 0.16a	0.65 ± 0.12a	0.59 ± 0.13a	0.67 ± 0.12a
Tribenuron-methyl + Tebuconazol(1:1 toxicity)	0	3.23 ± 0.75a	2.79 ± 0.77a	3.04 ± 0.74a	2.96 ± 0.57a	3.20 ± 0.84a	0.64 ± 0.13a	0.70 ± 0.12a	0.68 ± 0.22a	0.64 ± 0.13a	0.67 ± 0.14a
	0.025LC_50_	3.05 ± 0.73a	3.06 ± 0.82a	2.79 ± 0.57a	3.21 ± 0.75a	3.24 ± 0.94a	0.72 ± 0.15a	0.62 ± 0.14a	0.74 ± 0.15a	0.62 ± 0.12a	0.75 ± 0.15a
	0.05LC_50_	3.23 ± 0.73a	3.22 ± 0.68a	3.12 ± 0.68a	3.08 ± 0.85a	3.04 ± 0.73a	0.65 ± 0.14a	0.82 ± 0.21a	0.66 ± 0.15a	0.70 ± 0.15a	0.85 ± 0.16a
	0.1LC_50_	3.44 ± 0.73a	3.47 ± 0.94a	2.89 ± 0.58a	3.16 ± 0.83a	3.25 ± 1.04a	0.84 ± 0.23a	0.91 ± 0.22a	0.74 ± 0.14a	0.73 ± 0.21a	0.69 ± 0.12a

## Discussion

Both acute and chronic toxicity tests play an important role in the risk evaluation of pesticides to earthworms and are considered valuable for predicting the responses of soil organisms to pesticides^5,28,29^. In all acute toxicity test methods, only contact filter paper and artificial soil tests adopt mortality (LC_50_) as the toxic endpoint, and thus have received a significant attention in the toxicological studies^29,30^. Particularly, artificial soil test represents to some extent the natural conditions, and thus it is recommended as a standard method by researchers and several research organizatios^29,31–33^. In this study, as per the toxicity classification standard of OECD^31^, we report low toxicity of TBM to the soil earthworms using both toxicity methods, which is consistent with the fact that TBM almost nontoxic to reptiles^34^. However, both methods revealed different level of acute toxicity of TEB (Table 1). The LC_50_ of TEB obtained from contact filter paper test demonstrated that TEB is highly toxicity to the earthworm. Contrarily, artificial soil test demonstrated low toxicity of TEB to the earthworm. Our results are partly in line with findings of Wang *et al*.^29^ who also reported differences in TEB toxicity to earthworm in the contact filter paper and artificial soil tests. Meanwhile, our results also indicated that TEB has relatively greater contact toxicity than that of TBM. The low toxicity of TEB to earthworms in the artificial soil might be due to the fact that TEB can be easily adsorbed by the soil particles, which nevertheless reduces its bioavailability, exposure and toxicity to earthworm in the soil environment^35^. Apart from individual effects, the combined effects of two or more pesticides could be either greater or less than that predicted ones depending on individual or combined dose, pesticides chemistry, and organisms metabolic response^36–38^. In this study, both TBM and TEB in mixture exhibited antagonistic toxicity to earthworms in the both acute test methods (Table 1). The observed antagonistic effect could be due chemical interactions of TBM and TEB with each other that ultimately reduced their combined effect on the earthworms. However, underlying mechanisms of TBM and TEB interactions, and possibly the biochemical basis of the reduced effect of pesticide mixture on earthworm still need further definition.

In the chronic toxicity test, we investigated the impact of individual and pesticides mixtures on biochemical and genetic properties (DNA) of the earthworms in the natural soil. The SOD-CAT antioxidant system scavenges free radicals generated in phase I, and thus fight against oxygen damage^39^. In individual pesticide exposure experiments, the SOD activity increased significantly under medium concentration (0.05 LC_50_) of TBM but decreased significantly under high concentration (0.1 LC_50_) on day 3. Contrarily, the SOD activity increased significantly under high concentration (0.1 LC_50_) of TEB until on day 7. Similarly, the effect time of TEB on CAT activity in the earthworms was also longer than that of TBM; a significant increase in the CAT activity lasted for three and fourteen days at high concentration of TBM and TEB, respectively. Overall, these results indicated that the effect of TEB on earthworms is bigger than that of TBM under the same acute exposure concentrations, which is in line with our results from acute test experiments. Generally, the elevated SOD and CAT activities are considered as a direct response to the enhancement of superoxide anion radicals whereas reduced SOD activity could be due to the natural antioxidant defenses began to be overwhelmed^40,41^. On day 14 under exposure of either TBM or TEB, the SOD and CAT activities recovered to control levels, thus indicating that both pesticides did not cause serious oxidative damage to the soil earthworms. The difference in effect-time of pesticides exposures may be due to their different chemistry and mode of actions. Meanwhile, it is important to mention that TBM did not exhibit long persistence as compared to that of TEB in the soil. For example, previous study reported that the half-life of TBM in soil is 5.37 days^42^, whereas that of TEB is 74.2 days^19^. During the whole exposure time, MDA contents in the earthworms were not significantly affected under either TBM or TEB exposures except the MDA contents increased steadily under high dose of TEB on day 3, which further confirmed that the earthworms did not experience serious oxidative stress when exposed to the soil contaminated with TBM or TEB. The cellulase activity was significantly inhibited under high concentrations of TBM on day 3 and 7, and then recovered to the control level, thus implying that TBM can affect the biochemical metabolism of earthworms in earlier stage of exposure. That phenomenon can be attributed to the relatively shorter persistence of TBM in the soil environment, and that is why, as mentioned earlier, its negative effect on the earthworms disappeared with increasing exposure time. Similar to our results, previously the inhibitory effects of some herbicides such as acetochlor and fomesafen on the cellulase activity of soil earthworms are also reported^43,44^. Interestingly, the TEB did not change cellulase activity at all concentrations as compared to the controls during whole exposure period, which clearly indicates that TEB did not affect the earthworm’s ability to decompose cellulosic materials in soil. Combined toxicity of TBM and TEB showed that, like individual toxicity, the mixture of both pesticides only has a transitory effect to enzyme activity and lipid peroxidation in the earthworms at earlier exposure time, indicating that TBM and TEB can’t cause more severe oxidative damage to earthworms when they co-occurred in the soil. Finally, we tested genetic toxicity of pesticides in the earthworms using comet assay. We did not observe any significant difference in the comet length and olive tail moment at all concentrations of both pesticides, either used alone or as mixture, in whole exposure period, thus suggesting that pesticides exposure did not cause genetic toxicity (DNA damage) in the earthworms.

It is noted that we don’t have any information about the mode of action (additivity, synergy, and antagonism) of pesticides mixture in chronic toxicity experiment, therefore, we are unable to provide any logical explanation why pesticides mixture failed to induce initially assumed effect on earthworms. According to Hernández *et al*.^37^, interaction of pesticides to organisms does not occur in combined toxicity when the concentration was at or below adverse effect level. Therefore, the results from pesticides mixture experiment may be attributed to low concentrations used in this study. Actually, the concentrations of both pesticides adopted in this study were relatively higher than initial residues of TBM and TEB in natural soils reported by previous studies which reported that initial concentrations of TBM and TEB in soils under field conditions were about 0.3 and 0.7 mg kg^−1^ at 1.5 times of recommended dosage^42,45^. Thus, based on the results of this study, we suggest that both pesticides either alone as mixture in wheat rhizosphere are safe and acceptable if used in field at the recommended dose unless otherwise their ecological concerns are properly addressed with respect to other soil biota.

## Methods

### Pesticides and earthworm

Both pesticides TBM (purity 95.6%) and TEB (purity 97.5%) were of analytical grade and obtained from Hansen Biologic Science Co., Ltd., Qingdao, China. The earthworm *E*. *fetida* (weighing between 350 and 500 mg) with well-developed clitella was purchased from an earthworm culturing farm in Qingdao, China. The earthworms were maintained in the natural soil (mixed 5‰ decomposed cattle manure, 35% of moisture) at 20 ± 1 °C for at least two weeks prior to use in the experiments. Before toxicity experiments, earthworms were incubated for 24 h on moist filter paper at 20 ± 1 °C in the dark to empty their gut contents.

### Acute toxicity test on *E*. *fetida*

Both contact filter paper and artificial soil tests were performed following the OECD guideline^31^. For contact filter paper test, a 9 cm Petri dish was lined with a piece of filter paper without overlapping. The pesticides used alone or as mixture were dissolved in acetone and loaded on the filter paper (2 mL solution per dish). The control treatments were also run in parallel with acetone only. After the acetone was evaporated in an airing chamber, filter was remoistened with 2 mL of distilled water. One earthworm was placed on the dish, and each dish was covered with plastic lid with small holes and incubated in the dark at 20 ± 1 °C. The mortality was recorded on 24 and 48 h. Ten earthworms were set for each treatment. In the artificial soil test, soil consisted of 70% quartz sand, 20% kaolin clay, 10% sphagnum peat. We added a small amount of calcium carbonate to adjust soil pH at 6.0 ± 0.5 whereas distilled water was added to adjust the water content to 35%. About five-hundred grams of artificial soil were added into 1 L glass beaker, and the desired amount of pesticide solutions was thoroughly mixed into soil to allow a homogeneous distribution of the pesticides. Ten earthworms were placed into each beaker and were covered with plastic lid with small holes to allow aeration. The control soil treatments were prepared similarly but macrocosms contained only solvent. Each treatment was performed in three beakers. The earthworms were incubated at 20 ± 1 °C in a 12:12 h light-dark regime. The mortality was monitored on day 7 and 14 after application.

In each toxicity test, preliminary experiments were conducted to determine the range of concentrations that resulted in 0–100% mortality for each pesticide. Then, six test concentrations in a geometric series and a control were used to obtain the LC_50_ value of each pesticide. The combined toxicity of TBM and TEB were conducted at an equal-toxic ratio (1:1) based on the observed LC_50_ values, and the test method was same to the single acute toxicity test.

### Biochemical assays

The exposed concentrations were according to LC_50_ of acute toxicity of artificial soil test on day 14. For individual toxicity, the concentrations of TBM were set as 0 (Control), 0.025 LC_50_ (13 mg kg^−1^), 0.05 LC_50_ (26 mg kg^−1^), and 0.1 LC_50_ (52 mg kg^−1^), and the concentrations of TEB were set as 0 (Control), 0.025 LC_50_ (7 mg kg^−1^), 0.05 LC_50_ (14 mg kg^−1^), and 0.1 LC_50_ (28 mg kg^−1^). The concentrations of join toxicity of TBM and TEB were also conducted at an equal-toxic ratio (1:1) and were set as 0 (Control), 0.025 LC_50_, 0.05 LC_50_, and 0.1 LC_50_. But in this section, natural soil was used as natural habitat of the earthworms. The natural soil was collected from the surface (0–20 cm) in a Peony Garden (with no history of pesticide application) located in Qingdao Agricultural University, China. Its chemical properties were: pH 6.8, organic matter 25.3 g kg^−1^, available nitrogen 143 mg kg^−1^, available phosphorus 32 mg kg^−1^, and available potassium 168 mg kg^−1^. The exposed method of earthworm was same to acute toxicity of artificial soil test. Each treatment was in triplicate. After 3, 7, 14, 21, and 28 days of exposure, two earthworms were collected from each soil and rinsed with distilled water and then placed on the petri dish with moistened filter paper to purge their gut contents for 24 h. The gut-cleaned earthworms were transferred into a pre-chilled vitreous tissue homogenizer with 5 ml homogenization buffer (50 mM Tris pH 7.5, 250 mM sucrose, 1 mM DTT, and 1 mM EDTA). The homogenates were centrifuged at 4 °C for 20 min at 9000 g. The supernatants were collected to determine the enzyme activity, malondialdehyde (MDA), and protein content.

The protein concentration in supernatants was determined using dye-binding method described by Bradford^46^. The activity of SOD was assayed by measuring the inhibition of photochemical reduction of nitro blue tetrazolium as described by Durak *et al*.^47^. One unit (U) of SOD activity was defined as the quantity of enzyme that caused 50% inhibition of nitro blue tetrazolium, and the results were expressed as U mg^−1^ protein. The activity of CAT was determined following the method of Xu *et al*.^48^. One unit of CAT was defined as the enzyme amount required to degrade half of H_2_O_2_ at 25 °C in 100 s, and the results were expressed as U mg^−1^ protein. The activity of cellulase was measured using a carboxymethyl cellulose assay described by Ghose^49^, and the enzyme activity was expressed as mg glucose mg^−1^ protein h^−1^. The content of MDA was determined by thiobarbituric acid assay according to the method of Miller and Aust^50^ with the exception that amount of MDA formed was calculated using the absorbance coefficient 1.56 × 10^5^ M^−1^ cm^−1^.

### Extraction of earthworm coelomocytes and comet assay

In this assay, the experimental design of exposure concentration and sample time were same to the biochemical assay. The extraction of earthworm coelomocytes and comet assay were performed following the methods of Eyambe *et al*.^51^ and Singh *et al*.^52^, respectively with some modifications as described by Lin *et al*.^53^. The processed samples were viewed by a fluorescence microscope (Leica DM2500, Germany) equipped with a digital imaging system. For each sample, one hundred of non-overlapping cells were randomly selected to score, and the captured images were analyzed by CASP software^54^.

### Statistical analysis

All date were presented as mean ± standard deviation (SD) and analyzed by SPSS 18.0 software (SPSS, Chicago, USA). The probit analysis was performed to assess the acute toxicity of TBM and TEB on earthworms. The Marking additive index (AI) method^55^ was used to analyze the joint effects of TBM and TEB mixtures. The toxic summation of combined compound (S) was calculated by the equation S = Am/A + Bm/B, where A and B are medium lethal concentration (LC_50_) values of TEB and TEB alone, respectively. But the Am and Bm are LC_50_ values of the pesticides mixture. The AI can be calculated as follows: if S ≤ 1.0, AI = 1/S − 1, and if Så 1, AI = 1 − S. The joint toxicity was characterized as the following: if AI = 0, additive effect, if AIå 0, synergistic effect, and if AI < 0, antagonistic effect. For biochemical test and comet assay, one-way analysis of variance (ANOVA) was performed to determine statistical differences among treatments followed by the Dunnett *t*-test at a confidence level of 0.05.

### Data availability

The datasets generated in this study are available from the corresponding author on reasonable request.
